# Holistic Approach to Immune Checkpoint Inhibitor-Related Adverse Events

**DOI:** 10.3389/fimmu.2022.804597

**Published:** 2022-03-30

**Authors:** Remo Poto, Teresa Troiani, Gjada Criscuolo, Giancarlo Marone, Fortunato Ciardiello, Carlo Gabriele Tocchetti, Gilda Varricchi

**Affiliations:** ^1^ Department of Translational Medical Sciences, University of Naples Federico II, Naples, Italy; ^2^ Medical Oncology, Department of Precision Medicine, University of Campania Luigi Vanvitelli, Naples, Italy; ^3^ Center for Basic and Clinical Immunology Research (CISI), University of Naples Federico II, Naples, Italy; ^4^ Moscati Hospital Pharmacy, Aversa, Italy; ^5^ World Allergy Organization (WAO) Center of Excellence, Naples, Italy; ^6^ Institute of Experimental Endocrinology and Oncology (IEOS), National Research Council, Naples, Italy

**Keywords:** cancer, cytotoxic T lymphocyte-associated protein (CTLA-4), immunotherapy, immune checkpoint inhibitor (ICI), immune-related adverse event (irAE), programmed cell death protein -1 (PD-1), PD-L1

## Abstract

Immune checkpoint inhibitors (ICIs) block inhibitory molecules, such as cytotoxic T-lymphocyte-associated protein 4 (CTLA-4), programmed cell death protein 1 (PD-1), or its ligand, programmed cell death protein ligand 1 (PD-L1) and enhance antitumor T-cell activity. ICIs provide clinical benefits in a percentage of patients with advanced cancers, but they are usually associated with a remarkable spectrum of immune-related adverse events (irAEs) (e.g., rash, colitis, hepatitis, pneumonitis, endocrine, cardiac and musculoskeletal dysfunctions). Particularly patients on combination therapy (e.g., anti-CTLA-4 plus anti-PD-1/PD-L1) experience some form of irAEs. Different mechanisms have been postulated to explain these adverse events. Host factors such as genotype, gut microbiome and pre-existing autoimmune disorders may affect the risk of adverse events. Fatal ICI-related irAEs are due to myocarditis, colitis or pneumonitis. irAEs usually occur within the first months after ICI initiation but can develop as early as after the first dose to years after ICI initiation. Most irAEs resolve pharmacologically, but some appear to be persistent. Glucocorticoids represent the mainstay of management of irAEs, but other immunosuppressive drugs can be used to mitigate refractory irAEs. In the absence of specific trials, several guidelines, based on data from retrospective studies and expert consensus, have been published to guide the management of ICI-related irAEs.

## Introduction

**Figure 1:**
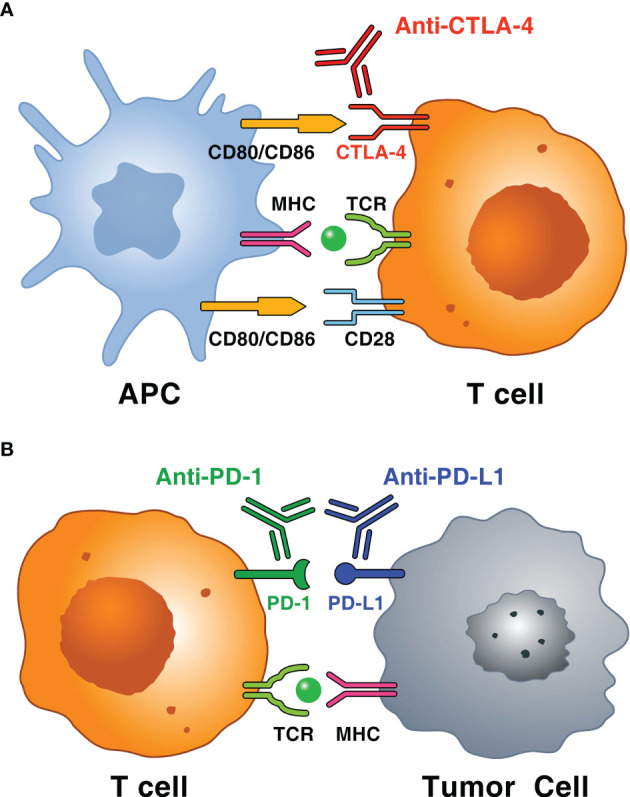
Schematic representation of immune mechanisms of immune checkpoints and immune checkpoint inhibitors (ICIs). **(A)** T cells, particularly CD4^+^ T cells in the lymph node, recognize tumor antigens in the context of MHC molecules or antigen-presenting cell (APC) and T cell receptor (TCR) on T cells. The interaction between CD80 (also known as B7-1) or CD86 (also known as B7-2) on APC and CD28 mediates T cell co-stimulation in conjunction with TCR signals. CTLA-4 on activated T cell interacts with both ligands (i.e., CD80 or CD86) with higher affinity and avidity than CD28 and, unlike CD28, sends an inhibitory signal to T cell. Monoclonal antibodies anti-CTLA-4 (i.e., ipilimumab) block this inhibitory pathway restoring T cell activity. **(B)** T cells, particularly cytotoxic CD8^+^ T cells, which recognize tumor antigens in the context of MHC class, result in the adaptive expression of PD-L1 on the surface of tumor cells. The interaction between PD-1 and PD-L1 negatively regulates the anti-tumor T cell response. This interaction is useful in preventing autoimmunity in physiological conditions, whereas cancer cells exploit this mechanism to escape from immune system upregulating PD-L1 expression. Anti-PD-1 (i.e., pembrolizumab, nivolumab and cemiplimab) and anti-PD-L1 mAbs (i.e., atezolizumab, avelumab and durvalumab) block this inhibitory pathway restoring T cell activity.

Immune checkpoint inhibitors (ICIs) have revolutionized the management of several advanced cancers (1, 2) and can result in durable responses in a percentage of patients (3–5). ICIs are monoclonal antibodies (mAbs) that block inhibitory molecules involved in regulation of immune system pathways, such as cytotoxic T-lymphocyte-associated protein 4 (CTLA-4) (e.g., ipilimumab), programmed cell death protein 1 (PD-1) (e.g., nivolumab, pembrolizumab, cemiplimab), or its ligand programmed cell death protein ligand 1 (PD-L1) (e.g., atezolizumab, avelumab, durvalumab) in the tumor microenvironment, which leads to systemic immune cell activation (**Figure 1**) (6, 7). Immune checkpoints constitute mechanisms of central relevance in the regulation of immune response to avoid autoimmunity and limit tissue damage (8, 9). Immune checkpoints can be exploited by cancer cells as mechanisms of immunoevasion and immunoresistance (10).

ICIs activate T cells and are often associated with a large spectrum of autoimmune responses, which are commonly referred to as “immune-related Adverse Events” (irAEs). The pleiotropic manifestations of irAEs can affect almost any organ (e.g., skin, colon, endocrine organs, joints, heart and lungs) and clinicians should be able to recognize and treat the heterogeneous manifestations of irAEs. Several comprehensive reviews have examined in detail the toxicity of ICIs affecting the skin (11–13), the gastrointestinal (14–16) and cardiovascular systems (17–24), the lung (25, 26), the endocrine organs (27–29), the joints (30, 31), the nervous (32) and the hematologic systems (33). In this review, we summarize the most recent observations and the complex pathophysiology and clinical characteristics of irAEs and their putative predictors and emerging therapies.

## Incidence/Prevalence of IrAEs

Distinct immunological mechanisms underlie anti-CTLA-4 (ipilimumab) and anti-PD-1/PD-L1 checkpoint blockade (34). Therefore, it is not surprising that the incidence of any irAEs with these two groups of ICIs varies greatly. The pattern, incidence and severity of irAEs vary according to the type of ICI (anti-CTLA-4 or anti-PD-1/PD-L1) and the treatment schedule (monotherapy or combination therapy). It has been estimated that the incidence of irAEs in patients treated with anti-CTLA-4 mAb (ipilimumab) is higher than in those treated with anti-PD-1/PD-L1 mAbs (35). The highest incidence and the high-grade irAEs are usually associated with combination therapy of ipilimumab plus anti-PD-1/PD-L1 (36). In a large meta-analysis examining 16,485 patients, colitis and hypophysitis were more frequent with ipilimumab, while diabetes and pneumonitis were more frequent with anti-PD-1/PD-L1 (35). Colitis, hepatitis, pancreatitis, and ICI-associated diabetes are more likely to be high-grade (37).

## Timing Of IrAEs

In melanoma patients treated with ipilimumab, the time of onset of skin-related irAEs is two to three weeks after ICI initiation, gastrointestinal and hepatic irAEs after six weeks, and endocrine irAEs after six to nine weeks (38, 39). Most high-grade irAEs resolve in two to five weeks with immunosuppression but some, such as arthritis, tend to persist (40). Endocrine irAEs (e.g., diabetes, thyroid dysfunctions) are usually irreversible and require prolonged hormone replacement therapy (39).

Fatal ICI-related irAEs tend to occur in the early phases of therapy and the incidence varies with the type of treatment. Fatalities are more common with combination therapy than with anti-PD-1/PD-L1 or anti-CTLA-4 (20). Fatality rates were approximately 39% for myocarditis and 5% for colitis (20).

There are some similarities between autoimmune manifestations of ICI-related irAEs and their spontaneous autoimmune counterparts but also several differences (41). For instance, ICI-induced diabetes can manifest with diabetic ketoacidosis, similar to T1D (42). The frequency of autoantibodies in ICI-induced diabetes is lower than in T1D (42, 43). ICI-induced hyperthyroidism is typically found at presentation and usually progresses to hypothyroidism (44, 45). ICI-induced colitis differs from inflammatory bowel disease (IBD) because is usually reversible (14).

## Long-Term Adverse Effects of ICIs

ICIs have been successfully introduced in the treatment of various cancers only a few years ago. Therefore, there is limited experience on the long-term side effects of ICIs. Acute irAEs have thus far attracted major attention owing to their dramatic clinical presentation and need for urgent treatment. However, increasing evidences indicate that chronic irAEs are more prevalent than originally recognized (46, 47). Endocrinopathies (such as ICI-induced hypothyroidism and diabetes) and rheumatological toxicities (such as arthritis) are the most common chronic irAEs (48, 49). Endocrinopathies provide classical examples of irreversible damage of the relevant hormone-secreting cells. These syndromes are usually irreversible and require the use of lifelong exogenous hormone replacement therapy (45, 50). On the other hand, ICI-induced arthritis provides a classical example of smouldering inflammation in which ICIs trigger persistent subacute or chronic arthritis, closely mimicking that of rheumatoid arthritis (48, 51).

Several experimental studies have demonstrated that CTLA-4 and PD-1/PD-L1 axes are critical negative regulators of atherosclerosis (52–55). A recent retrospective study by Drobni et al. reported an association between ICIs with accelerated progression of atherosclerosis and cardiovascular events (56). They found increased atherosclerotic inflammatory activity 5 months after ICI therapy (57). Another retrospective study on 20 patients with melanoma found by positron emission tomography/computed tomography with 2-[^18^F] fluorodeoxyglucose (^18^F-FDG) that ICI therapy induced inflammatory activity in large arteries (57). The results of these two studies will certainly influence the approach to cardiovascular care for individuals receiving ICIs. Cardiac evaluation before initiation of ICI treatment should focus on long-term prevention rather than focusing only on early irAEs. Moreover, cancer trials should prospectively examine not only early but also late cardiovascular events (58).

## Pathophysiology of IrAEs

**Figure 2:**
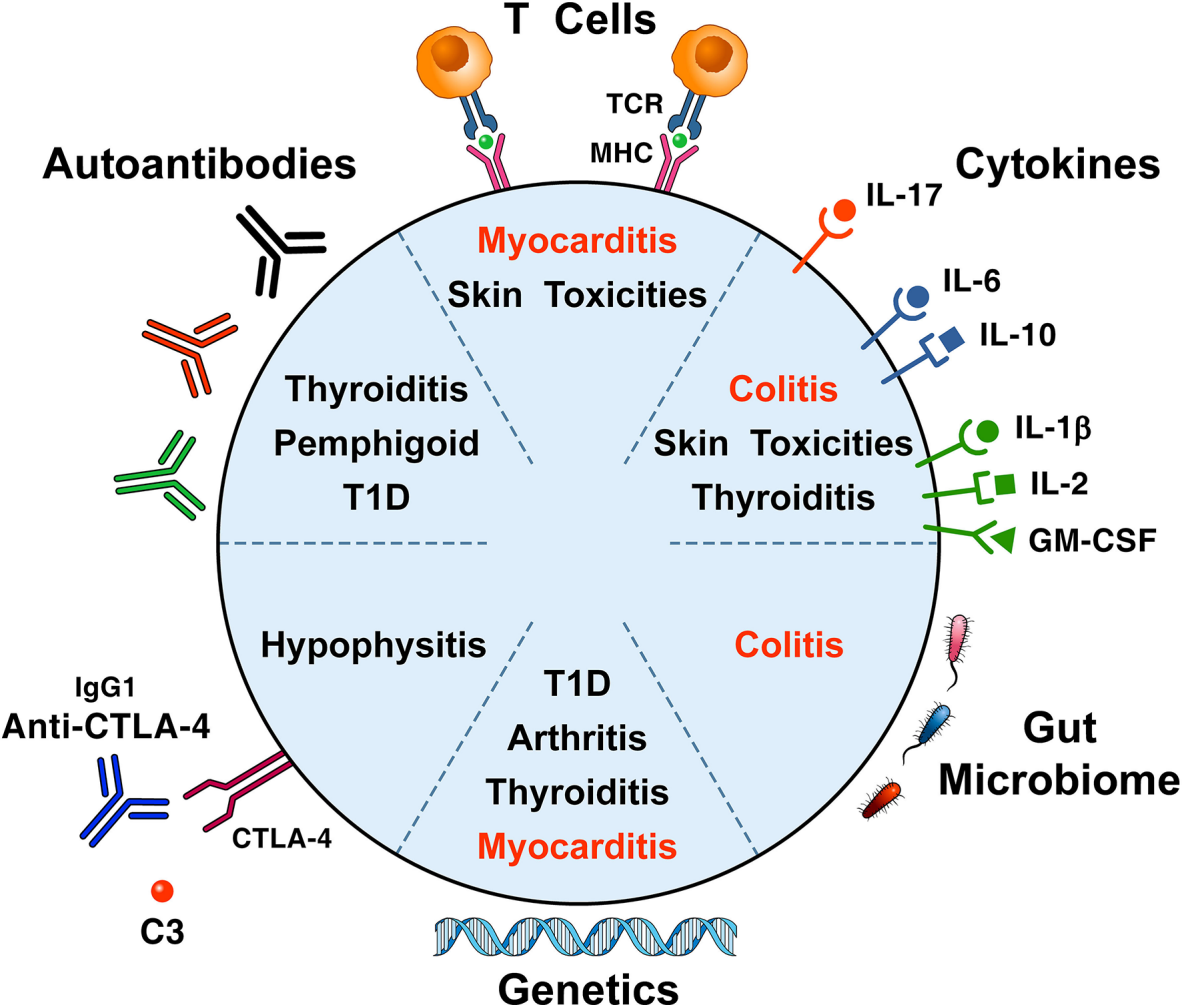
Proposed immunopathogenic mechanisms for the development of immune checkpoint-induced immune-related adverse events. A proposed mechanism postulates that self-antigens (e.g., heart and skeletal muscle antigens) activate T cell clones driving antitumor responses and organ-specific autoimmunity (24). Thyroid autoantibodies may be involved in patients who develop thyroid dysfunction (44, 59, 60), ICI-associated diabetes (42, 43), bullous pemphigoid (61), hypophysitis (62, 63), and myasthenia gravis (64). Cytokines/chemokines released from immune cells can cause immune-mediated tissue damage (12, 65, 66). The pivotal role of genetic factors in the development of ICI-associated irAEs, originally highlighted in mice (67, 68), has been confirmed in patients with arthritis (69), ICI-associated diabetes (42, 43, 70), and pruritus (71). There is growing evidence that gut microbiome may play a role in the development of experimental irAEs (72, 73) and of colitis in patients with melanoma (74, 75). irAEs contributing to most fatalities are presented in red.

Several immunopathogenic mechanisms (i.e., cellular autoimmunity, autoantibodies, complement activation, cytokines/chemokines release, genetics and alterations of the gut microbiome) have been suggested to be involved in the development of ICI-related irAEs (**Figure 2**).

### Cellular Autoimmunity

The importance of T cells in the mechanisms of ICI-associated irAEs is supported by genetic loss-of-function studies in mice (67, 68). Autoreactive T cells can be activated by shared antigens between tumor and peripheral tissues. Shared T cell clones in the tumor, heart, and skeletal muscle were found in melanoma patients, who died from fatal myocarditis and myositis after treatment with anti-CTLA-4/PD-1 mAbs (76). The exact mechanism of cardiac toxicity remains unknown, but it has been suggested that shared antigens may drive both antitumor responses and organ-specific autoimmunity (24). Shared T cell antigens were found in the skin and tumor of lung cancer patients who developed skin toxicities (77). Similarly, vitiligo is common in melanoma patients treated with ICIs (78). Recently, Lozano and collaborators demonstrated that in melanoma patients treated with anti-PD-1 or anti-PD-1 and anti-CTLA-4 combination, two pretreatment factors in peripheral blood - activated CD4 memory T cell abundance and TCR diversity - were associated with severe irAEs development (79).

CTLA-4 is a modulator of Tregs (80) and these cells act as gatekeepers for the prevention of autoimmunity. The role of Tregs in ICI-induced irAEs deserves additional studies (81). It has been suggested that tissue-resident memory T cells (Trm) in tumor microenvironment can play a role in irAEs (82). Single-cell analysis of ICI-associated colitis patient samples found expansion of CTLA-4^+^ Treg cells and differentiation of CD8 Trm cells to cytotoxic effector cells (83).

### Humoral Immunity

Anti-thyroglobulin (TG) and/or anti-thyroid peroxidase (TPO) autoantibodies are found in 13–70% of patients who develop ICI-related thyroid dysfunction (44, 59, 60, 84). Thyroid autoantibodies increase the risk of ICI-induced thyroid dysfunction (85–87) and β-cell autoantibodies are found in approximately 50% of patients with ICI-induced diabetes (42, 43, 88). Autoantibodies anti-BP180 can be found in the majority of patients with anti-PD-L1-associated bullous pemphigoid (61).

Human pituitary cells express CTLA-4 at both mRNA and protein levels and in an animal model the injection of anti-CTLA-4 antibodies induced lymphocytic infiltration and complement activation of the pituitary gland (62). Anti-pituitary antibodies were detected only in patients with ipilimumab-associated hypophysitis but not in those without hypophysitis (62). Autoantibodies against guanine nucleotide-binding protein G subunit alpha (GNAL) and integral membrane protein 2B (ITM2B) have been found associated with ICI-induced hypophysitis (63). These findings implicate both autoantibodies and T cell-mediated processes in ICI-associated pituitary destruction (89). Overexpression of pituitary CTLA-4 was reported in a patient with severe ipilimumab-associated hypophysitis (90). It has been suggested that hypophysitis is caused by complement activation from endogenous autoantibodies and/or exogenous IgG1 anti-CTLA-4 (ipilimumab) (62). IgG1, used in ipilimumab, activates the classic complement pathway explaining the elevated frequency of pituitary gland damage compared with its occurrence in patients treated with anti-PD-1/anti-PD-L-1 IgG4 antibodies (91, 92).

Anti-acetylcholine receptor antibodies can be found in approximately 50% of patients with ICI-induced myasthenia gravis (64). Patients with ICI-induced arthritis are commonly rheumatoid factor (RF) and cyclic citrullinated peptide (CCP) negative (69). Unfortunately, in the majority of these studies the presence of autoantibodies prior to ICI initiation has not been evaluated.

### Cytokines and Chemokines

Cytokine release syndrome (CRS) is a systemic inflammatory disorder characterized by a massive release of cytokines (93). It can present with a variety of symptoms ranging from mild (e.g., fever, fatigue, nausea, rash) to life threating, sometime fatal. A recent analysis of WHO global pharmacovigilance database, found that ICI-related CRS can occur in ICI-treated patients (94). On similar ground, cytokines and chemokines are involved in different irAEs. Increased baseline IL-17 concentrations were temporally associated with subsequent development of ICI-associated colitis (65). Increased IL-6 and IL-10 concentrations were found in patients with skin irAEs (12). Increased concentrations of IL-1β, IL-2, and GM-CSF at baseline have been associated with ICI-related thyroid dysfunction (66). High concentrations of T cell chemotactic chemokines (i.e., CXCL9 and CXCL10) are associated with irAEs (95). A recent study in patients with melanoma treated with combined immune checkpoint blockade (CICB) targeting CTLA-4 and PD-1 who developed ≥ grade 3 colitis demonstrates an intestinal overexpression of *IL1β* and *TNF* compared to normal tissue (96).

### Genetic Factors

Genetic factors influence the development and progression of several autoimmune disorders. The importance of genetic factors in the development of ICI-associated irAEs was originally highlighted by genetic loss-of-function studies in mice (67, 68). Thus, the possibility exists that genetic susceptibility may play a role in the pathogenesis of irAEs. Experimental studies have demonstrated that CTLA-4 and PD-1 deletion or inhibition can cause autoimmune myocarditis with lymphocytic infiltration of cytotoxic T-cells (67, 97–99). The majority (≅ 52%) of patients with ICI-related arthritis possess the RA-associated HLA-DR susceptibility allele (69). The majority of patients with ICI-related diabetes had at least one HLA-DR risk allele (70). HLA-DR4 predominance has been reported in patients with ICI-induced diabetes (42, 43). HLA-DRB1*04:05 has been associated with ICI-induced arthritis (69) and HLA-DRB1*11:01 with pruritis (71). A multicenter study found that a polygenic risk score (PRS) for thyroid disorders is associated with developing thyroid irAEs in patients with non-small cell lung cancer (NSCLC) treated with anti-PD-1 or anti-PD-1 and anti-CTLA-4 combination (84). In this study, thyroid irAEs were associated with better response to ICIs. Moreover, in a phase 3 randomized controlled trial (RCT), it was found that PRS for dermatological autoimmune diseases were associated with increased risk for skin irAEs and longer overall survival in bladder cancer patients treated with atezolizumab (100).

### Microbiome

The multiple interactions among tumor microenvironment, microbiome, host factors, and response to ICI, and the development of ICI-associated irAEs are largely unknown (101). There is evidence that the gut microbiome might play a role in tumor response (102–104). For instance, gut microbiome modulates response to anti-PD-1 in melanoma patients (105) and epithelial tumors (103). Mice repleted with *B. fragilis* less likely developed irAEs after exposure to anti-CTLA-4 inhibitors (72). Moreover, microbiota-derived peptides from *Bacteroides* induced autoimmune myocarditis (73).

Melanoma patients treated with ipilimumab and with baseline gut microbiome enriched for *Faecalibacterium* and other *Firmicutes* had longer progression-free survival and overall survival (74). In a prospective study of melanoma treated with ipilimumab, patients with abundant *Bacteroidetes phylum* less likely developed ICI-induced colitis (75). A recent study found that gut microbiota signatures are associated with irAEs to CICB targeting CTLA-4 and PD-1 in melanoma patients and in experimental models (96). In this study, the rate of any grade of irAEs was high (93.5%) and 49% of patients experienced severe (≥ grade 3) irAEs. The alpha diversity of the gut microbiome in patients who did or did not develop severe irAEs was similar. However, *Bacteroides intestinalis* (*B. intestinalis*) were more abundant in patients with ≥ grade 3 irAEs *versus* those who did not. In melanoma patients who developed colitis there was an overexpression of mucosal *IL1β* and *IL-17*, but not *TNF*. These fascinating results were corroborated by results in experimental models in which mice, gavaged with different strains of *B. intestinalis* following gut sterilization with antibiotics, showed overexpression of *Il1b.* Moreover, fecal microbiota transplant (FMT) in antibiotic-treated animals using fecal material from human donors harboring high endogenous levels of *B. intestinalis* induced ileal overexpression of *Il1b* after administration of CICB. Collectively, these human and experimental studies highlight a contribution of commensal microbiota to intestinal damage associated with CICB.

## Putative Biomarkers of Immune-Related Adverse Events

Several studies have or are evaluating the possibility of identifying biomarkers of irAEs associated with different types of ICIs. **Table 1** lists genetic, clinical, immune, microbial and tumor biomarkers that have been linked to irAEs. In particular, some studies have identified an association between HLA and irAEs. The association of baseline antibodies (85–87, 109, 110), baseline cytokine levels (65, 66, 111, 112), and immune cell changes (79, 83, 113–116) suggests that humoral and cellular immunity play a role in some specific irAEs. In particular, activated CD4 memory T cell abundance and TCR diversity in peripheral blood are associated with severe irAEs development in patients with melanoma (79). The emerging results from parallel human and experimental models highlight a contribution of specific gut microbiota to the development of intestinal irAEs (74, 75, 96). It should be emphasized that the small size of these studies requires validation in larger cohorts of patients with different types of cancer.

**Table 1:** Putative predictors of immune adverse events associated with immune checkpoint inhibitors.

Genotype	HLA-DR4 association with ICI-associated diabetes in patients with a variety of cancers treated with anti-PD-1/PD-L1 (42). HLA-DR4 association with ICI-associated diabetes in patients treated with anti-PD-1/PD-L1 (43). HLA-DRB1*04:05 association with ICI-induced arthritis in patients with a variety of cancers (69). HLA-DRB1*11:01 association with ICI-induced pruritis and HLA-DQB1* 03:01 and colitis in patients with non-small lung cancer (NSCLC) or melanoma treated with anti-PD-1, anti-CTLA-4 or their combination (71).
Pre-existing autoimmune disease	irAEs are more frequent and occur sooner in patients with autoimmune disease treated with anti-PD-1 (106, 107). Pre-existing autoimmune disease associated with modest increases in hospitalization with irAEs in patients treated with ICIs (108).
Baseline autoantibodies	Thyroid autoantibodies (anti-TPO, anti-tg) at baseline increases the risk of thyroid dysfunction in patients treated with nivolumab or pembrolizumab (85–87, 109). Baseline autoantibody signatures, such as those targeting TNF-α signaling pathways may be predictive of irAEs in patients with melanoma treated with anti-CTLA-4, anti-PD-1 or their combination (110). Skin irAEs may be more frequent in patients with positive RF at baseline in patients with NSCLC treated with nivolumab or pembrolizumab (87).
Baseline cytokine levels	Baseline IL-17 serum levels may predict ICI-induced colitis in patients with melanoma treated with ipilimumab (65). Baseline IL-6 serum levels were associated with higher risk of toxicity in melanoma patients treated with ipilimumab (111). Cytokine toxicity score predictive of severe irAEs in patients with melanoma treated with ipilimumab, anti-PD-1 or their combination (112). Baseline serum levels of IL-1β, IL-2, and GM-CSF predict thyroid dysfunction in patients with a variety of cancers (66).
Immune cell changes	Reduction in circulating B cells, increase in CD2^lo^ PD-1^+^ B cells and plasmablasts precede adverse events in patients with melanoma treated with ipilimumab, anti-PD-1 or their combination (113). Neutrophil-to-lymphocyte ratio and platelet-to-lymphocyte ratio may predict appearance of irAEs in patients with NSCLC treated with anti-PD-1/PD-L1 (114). High baseline absolute eosinophil count (AEC) (> 135/μl) correlates with the risk of irAEs in patients with melanoma, renal cell carcinoma, and NSCLC treated with anti-CTLA-4 (115, 116). High proliferative index in circulating effector and control memory CD8^+^ T lymphocytes at early time points in melanoma patients treated with CICB who developed ≥ grade 3 irAEs (96). Lower expression of surface CD28 and CD27 on circulating CD4^+^ and CD8^+^ effector T lymphocytes of melanoma patients treated with CICB who did not develop severe irAEs (96). Increased activated CD4 memory T cells and TCR diversity in peripheral blood are associated with severe irAEs in patients with melanoma treated with anti-PD-1 or anti-PD-1 and ipilimumab combination (79).
Microbiome	*Bacteroidetes phylum* may be protective for development of colitis in melanoma patients treated with ipilimumab (75). *Faecalibacterium* may be predictive of colitis in melanoma patients treated with ipilimumab (74). *Bacteroides intestinalis* is associated with ≥ grade 3 colitis in patients with melanoma treated with combined ipilimumab and PD-1 blockade (96).
Tumor burden	High tumor burden is associated with higher risk of severe irAEs in patients with NSCLC (117).

## Clinical Manifestations of ICI Associated Autoimmunity

**Figure 3:**
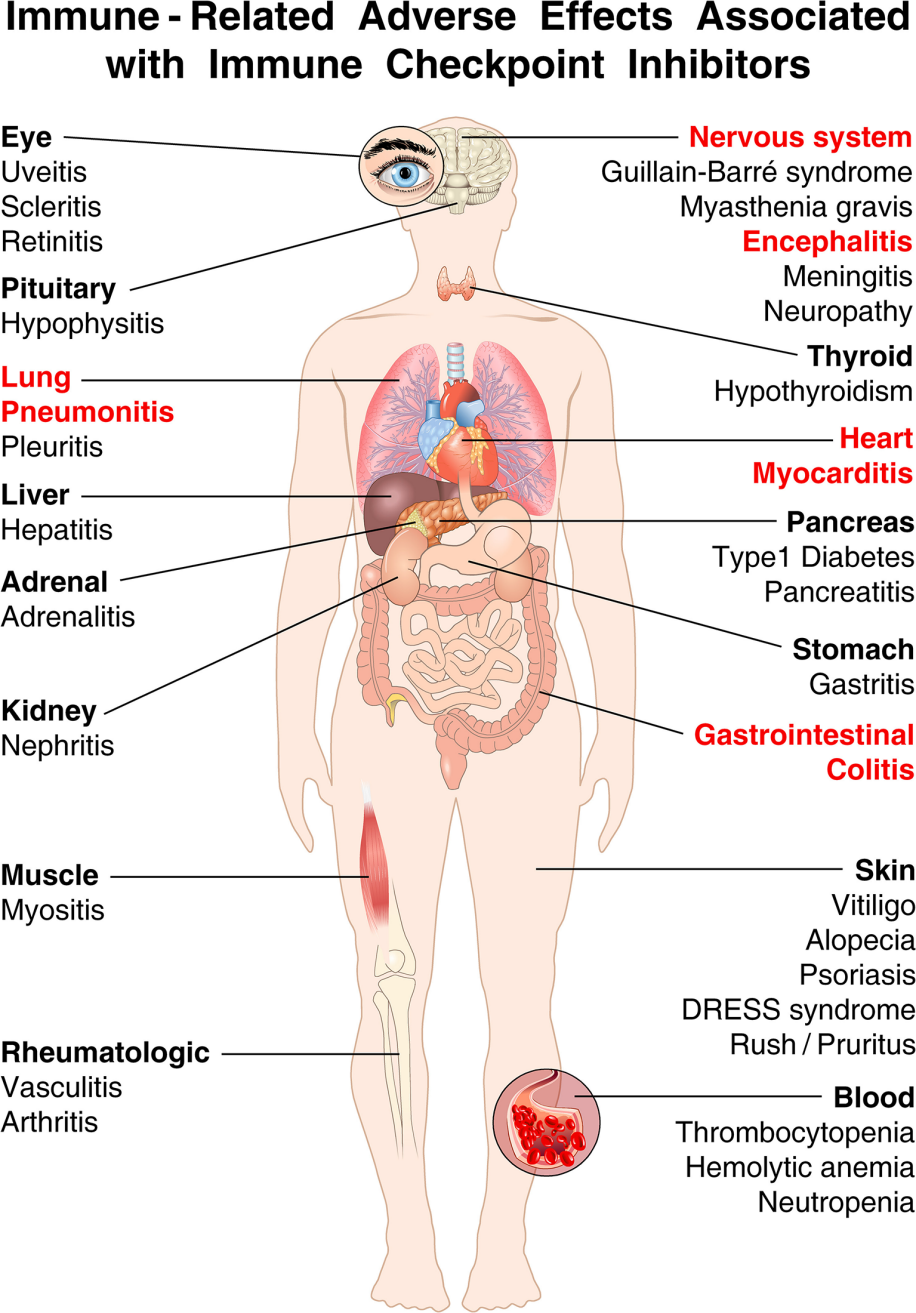
The spectrum of organs affected by irAEs associated with immune checkpoint inhibitors (ICIs) is very broad. Shown are the most common immune-related adverse events (irAEs) that clinicians can encounter in cancer patients treated with ICIs. irAEs contributing to most fatalities are highlighted in red [modified from (19)]. ICI-associated diabetes is almost exclusively seen in patients treated with anti-PD-1/PD-L1 antibodies, and rarely with ipilimumab monotherapy (42, 118). By contrast, hypophysitis occurs more often in patients receiving ipilimumab (119). Colitis occurs more commonly with ipilimumab and with combined immune checkpoint blockade than with anti-PD-1/PD-L1 alone (112, 120). Endocrinopathies (such as hypothyroidism, hypophysitis, and adrenal insufficiency) and rheumatological disorders have the highest incidence of development into subacute/chronic toxicity (49). Endocrine toxicities, unlike many other irAEs, are not managed using high-dose glucocorticoids, which have no effect on either initial severity and resolution (121, 122). Although acute myocarditis was the first cardiovascular irAEs associated with ICIs (123), an important unanswered question relates to the long-term cardiovascular sequelae of ICIs (49, 56, 57).

The results of a meta-analysis of 35 trials demonstrated the extreme heterogeneity of manifestations and severity of autoimmune complications of ICIs (35). **Figure 3** illustrates that irAEs can affect nearly every organ in association with ICIs.

### Skin Rash

Skin manifestations during ICI therapy show the highest incidence of irAEs and appear higher in patients with melanoma than in patients with other malignancies (124). ICI-related skin manifestations include maculopapular, eczematous, psoriasiform, lichenoid, and bullous eruptions, frequently associated with pruritus (125). Vitiligo associated with anti-PD-1 therapy differs from canonical vitiligo because of a patchy distribution, occurring in sun-exposed areas, and lacking the Köebner phenomenon (126). Bullous skin disease tends to occur in patients treated with anti-PD-1/PD-L1 rather than ipilimumab (127). Rare cases of severe Stevens-Johnson syndrome/toxic epidermal necrolysis have been reported (128).

Most patients have mild skin reactions, while severe (grade 3-4) toxicities are found in a low proportion of patients (5.8% for the ipilimumab + nivolumab combination, even lower values for anti-PD-1 monotherapy), significantly lower than the toxicities of other sites (such as hepatic or gastrointestinal) (129). The management of skin toxicities needs specific treatment for the type and severity of the condition: topical glucocorticoids are effective in treating low-grade skin reactions, whereas high-grade events must be treated with systemic glucocorticoids.

### Diarrhea and Colitis

Diarrhea is the most frequent manifestation of gastrointestinal toxicity from ICI and is one of the main causes of emergency department visits for patients treated with ICI (130). Colitis occurs more commonly with ipilimumab and with CICB than with anti-PD-1/PD-L1 mAbs alone (120, 131). No differences have been described in the incidence of diarrhea/colitis in different types of malignancy (132). The presence of ulcerative lesions appears to be related to a greater probability of glucocorticoid resistance and greater severity of diarrhea (133). Lesions primarily affect the distal colon, but lesions can occur in more proximal tracts. Colitis-associated autoantibodies are seldom present (134). Prophylactic budesonide is not effective against ipilimumab-induced colitis (135). Infliximab induces a shorter time to symptom resolution than glucocorticoids (136). Vedolizumab (an antibody directed against α4-β7 integrin), has also been used as glucocorticoid sparing agent (137). There is some evidence that early treatment with either infliximab or vedolizumab reduces symptom duration and glucocorticoid administration (138). Several clinical trials are evaluating the safety and efficacy of infliximab (NCT05034536, NCT03293784, NCT04407247) or certolizumab (NCT03293784) in clinical response of ICI-induced colitis.

Recent clinical and experimental models indicate that higher abundance of *B. intestinalis* is associated with high grade-colitis in melanoma patients treated with CICB (96). The gut microbiome appears to mediate intestinal toxicity *via* IL-1β and treatment of mice with an anti-IL-1R antagonist (anakinra) reduced intestinal inflammation. A clinical trial is evaluating the safety and efficacy of anakinra on irAEs and cytokine profiles in patients with different cancers (NCT04576429).

### Hepatitis and Pancreatitis

Immune-mediated liver injury caused by ICIs can present with fatigue, fever, nausea, and jaundice. The severity of hepatic toxicity can be classified in relation to the increase in liver enzymes (AST and ALT) and bilirubinemia. The incidence of irAEs varies between 3% to 9% for anti-CTLA-4, and 0.7% to 1.8% for anti-PD-1/PD-L1 (139). CICB (ipilimumab + nivolumab) is associated with an incidence of any-grade hepatotoxicity of 29% and high-grade hepatotoxicity of 17% (39). Approximately 50% of the patients with ICI-associated liver injury have antinuclear antibodies and 19% have anti-smooth muscle antibodies (140). Pancreatitis can also occur in response to ICIs (141).

### Thyroiditis

Thyroid dysfunction is the most common endocrinologic irAEs due to ICIs (27, 29, 45). The median time of onset is approximately 6 weeks after the start of immunotherapy. ICI-associated thyroid dysfunction is more common after anti-PD-1/PD-L1 antibodies or combination therapy than with ipilimumab alone (41, 142, 143). Patients receiving ICIs should undergo regular thyroid testing (i.e., TSH and T4). Patients usually experience a first transient phase of hyperthyroidism, followed by euthyroidism or hypothyroidism (45, 144). The presence of thyroid autoantibodies (anti-TPO and anti-TG) before ICI treatment increases the risk of thyroid dysfunction in patients treated with nivolumab or pembrolizumab (85–87, 109). The mechanism responsible for ICI-induced thyroid dysfunction is unclear. It has been hypothesized that polymorphic variants of the *PDCD1* in some individuals might predispose them to an increased risk of thyroid dysfunction (145). It is also unknown whether thyroid autoantibodies are the cause of thyroid dysfunction or the result of an immunological response to thyroid antigens released during ICI-related thyroiditis (146). ICI therapy can be continued with close follow-up and monitoring of TSH and T4 in the context of thyroiditis. In the presence of more severe irAEs, ICI should be held until symptoms resolve. Glucocorticoids do not improve the clinical course of thyroid dysfunction (121).

### Hypophysitis

Hypophysitis is a specific complication of ipilimumab treatment and rarely occurs in anti-PD-1/PD-L1-treated individuals (62, 91, 92). Most patients present with headache and/or fatigue (147, 148). Magnetic resonance imaging (MRI) of the brain highlights an expansion of the pituitary gland and/or infundibulum (147–149). Enlargement on MRI precedes the clinical diagnosis of ipilimumab-related hypophysitis (147). The pituitary gland decreases in size over 4-12 weeks leading to atrophy (147, 150, 151). Importantly, a normal MRI does not completely rule out hypophysitis, and therapy should be based on clinical symptoms and pituitary hormone levels (152). Glucocorticoids do not improve the degree or duration of hypophysitis (149). Hormone deficiencies are managed with the corresponding hormone replacement (149).

### Diabetes

Although not very common (42), PD-1 pathway blockade can cause autoimmune ICI-induced diabetes mellitus, which usually (≅ 70%) presents as diabetic ketoacidosis (29, 50, 153–156). ICI-associated diabetes rarely occurs in patients treated with ipilimumab (42, 118). In these individuals, C-peptide levels are very low and approximately 50% of patients have T1D associated antibodies (anti-glutamic acid decarboxylase) (43). Human leukocyte antigen risk alleles (HLA-DR4, -DQ8, -DR3, and DR2) can be associated with high frequency of spontaneous T1D (157). In ICI-induced diabetes, there is a predominance of HLA-DR4, which is present in approximately 70% of patients. Other HLA alleles associated with high risk of spontaneous T1D are not overrepresented in ICI-associated diabetes (42, 43). ICI-induced diabetes tends to be permanent (50) and attempts with glucocorticoids administration showed no recovery (158).

### Pneumonitis

Early clinical trials and meta-analyses suggested an incidence of ICI-associated pneumonitis of 3-5% (159–163); recent studies examining real-world populations suggest this could be as high as 13-19% (164–166). While the incidence of all-grade pneumonitis appears to be higher in the real-world population as opposed to clinical trials, the percentage of ≥ grade 3 pneumonitis appears to be relatively consistent across both populations (163–165, 167).

Immune-related pneumonia represents one of the main causes of death during treatment with anti-PD-1/PD-L1 alone and the fourth cause during combined treatment with ipilimumab plus anti-PD-1/PD-L1 (14% of total cases) after colitis, myocarditis and hepatitis (20). Anti-CTLA-4 treatment causes a lower incidence of immune-related pneumonia compared to treatment with anti-PD-1/PD-L1 alone (168). Immune-related pneumonia are usually associated with CICB (169). One-third of these patients are asymptomatic, whereas the others present with dyspnea and/or cough (159). Radiographic findings on chest computed tomography (CT) do not highlight specific characteristics (159, 170). Previous thoracic radiotherapy and previous lung disorders are predictors of pneumonitis associated with anti-PD-1 (171, 172). The differential diagnosis of these patients should be made after ruling out other causes of similar lung involvement. This issue is particularly relevant during the current outbreak of COVID-19 (173). Guidelines on the management of ICI-related pneumonitis have been published from ESMO (174) and ASCO/NCCN (175–177). A clinical trial is evaluating the safety and efficacy of infliximab *versus* intravenous immunoglobulin therapy (IVIG) in treating glucocorticoid-refractory pneumonitis (NCT04438382).

### Arthritis

Arthritis is not very common (≅ 4%) in patients with ICIs (178). A systematic review encompassing 372 patients found that the time of onset of arthritis ranged from 1 day to 53 months (median time: 4 months) (51); 49% had polyarthritis, 17% oligoarthritis, 10% arthralgia, and 21% polymyalgia rheumatica (178–181). More than half of patients had a “rheumatoid arthritis-like” presentation (51). RF and anti-citrullinated peptide antibodies (ACPA) are present in ≅19% of patients (51, 180, 182). Treatment of irAEs should be guided by severity (183). Most patients can be managed with non-steroidal anti-inflammatory drugs or intra-articular glucocorticoid injections. More severe patients, especially those who received CICB, require systemic glucocorticoids (182). Arthritis often persists even after stopping ICIs and may require prolonged immunosuppression with biological disease-modifying anti-rheumatic drugs (DMARDs) (40). Diagnostic and management algorithms for rheumatoid irAEs have been recently proposed (183).

### Myositis

ICI-related myositis can manifest in the form of acute or subacute myalgia or muscle weakness (183, 184). When concomitant myocarditis and myasthenia gravis-like symptoms (e.g., ptosis and oculomotor weakness) occur, fatality rates are relatively high (185). Muscle biopsy shows inflammation and myonecrosis (184). Myositis-associated antibodies (anti-TIF1-y, SRP, Ro52; PL-7, PL-12, or SRP) can be detected in ICI-associated myositis (186). Anti-striated muscle antibodies can be found even without clinical evidence of myasthenia gravis (187, 188). Glucocorticoids are the first-line therapy for ICI-associated myositis. Initial dosing can range from 0.5 mg/Kg prednisone daily up to 2,000 mg IV methylprednisolone (183). IVIG and plasmapheresis have been used in refractory cases (183, 184, 186).

### Myocarditis

The true incidence of ICI-associated myocarditis remains uncertain. Early ICI-based cancer trials did not prospectively screen for myocarditis (189). Moreover, because the diagnosis of myocarditis can be difficult, cases could easily be missed. Recent reports suggest that the incidence of ICI-associated myocarditis is 0.27% to 1.14% (76, 190). Myocarditis is an infrequent, but often lethal complication of ICI therapy (76, 191). Elevated troponin level and abnormal electrocardiogram were found in the majority of these patients with ICI-associated myocarditis. Interestingly, half of these patients showed preserved ejection fraction (190). The clinical manifestation of ICI-associated myocarditis is variable. Fulminant cases characterized by early-onset have been described (19, 123, 192). In these cases, cardiac arrhythmias are common (23, 190). The association of skeletal myositis and myasthenia gravis following ICI therapy should orientate for myocarditis (185, 193). “Smouldering” cases of myocarditis have been also reported (194).

Diagnosis of ICI-associated myocarditis is challenging and includes a combination of biomarker tests (troponin), cardiac MRI, late gadolinium enhancement, and possibly biopsy (T cell infiltrate) (195). Major adverse cardiac events (MACE) can occur also in patients with preserved ejection fraction. A troponin T level ≥ 1.5 ng/mL was associated with a marked increase in MACE during follow-up (190). The precise mechanisms by which ICIs cause cardiotoxicity remain undefined. Existing data support T cell-mediated immunity as a major component in pathogenesis, but many fundamental questions remain (24). Early and aggressive treatment with high doses of glucocorticoids is critical (132, 190, 196). Treatment of ICI-associated myocarditis includes ICI discontinuation, supportive management, and glucocorticoids (175). Prednisone (0.5 to 2.0 mg/kg), followed by 4–6 week taper upon symptoms improvement, is recommended (24, 175, 197). Despite this treatment, mortality remains substantial, and individual case reports demonstrate successful treatment with alemtuzumab (anti-CD52 mAb) (198), or abatacept (a fusion protein composed of the extracellular domain of CTLA-4 and the Fc region of human IgG1) (1). Prospective clinical trials are needed to compare the safety and efficacy of different immunosuppressive therapies in ICI-associated myocarditis. Recent experimental data point to the hypothesis that anti-PD-1 therapy induces a smouldering disruption of cardiac immunity towards an inflammatory phenotype, with manifest consequences on cardiac function in the presence of a second hit, in the form of systemic stress induced by presence of a tumor (199). This also indicates the possibility that the inflammatory phenotype raises the risk for the development of myocarditis upon exposure to additional, yet unknown risk factors (200). Furthermore, treatment with anti-PD-1 may first produce only a latent inflammatory involvement associated to dysregulated cardiac metabolism that may progress to overt myocarditis in a subset of patients (200, 201).

### Neurological

Neurological complications of ICIs (headache, myasthenia gravis, peripheral neuropathy, meningitis, and encephalitis) are uncommon (≅ 1%) (32, 77, 184, 202, 203). Myasthenia gravis (77) and encephalitis are more common with anti-PD-1 antibodies, whereas Guillain Barré and meningitis are more common with ipilimumab. MG associated with myositis and myocarditis has a poor prognosis (64, 203). Approximately 50% of MG patients have anti-acetylcholine receptor antibodies (64). Glucocorticoids and, in some patients, IVIG, are the mainstay of therapy (64).

### Hematologic

In contrast to other anticancer therapies, hematological irAEs in patients treated with ICIs are uncommon. Neutropenia, autoimmune hemolytic anemia, and immune thrombocytopenia can occur rarely (≅ 5%) (204, 205). A positive direct antiglobulin test is present in the majority (≅ 60%) of patients with ICI-related autoimmune hemolytic anemia (206). Glucocorticoids are the first line of therapy; IVIG or rituximab can be considered in difficult cases. Neutropenic patients can be treated with G-CSF (204, 206).

### Renal

Renal dysfunction is rare with ipilimumab and with anti-PD-1/PD-L1 therapies occurring in <1% of patients (207). The incidence is higher with combination of ipilimumab plus anti-PD-1/PD-L1 reaching approximately 4% (208, 209). Renal dysfunction is usually due to acute interstitial nephritis (210) or, more rarely, to glomerulonephritis (211).

### Ocular

irAEs of the eye are rare and occur in <1% of patients treated with ICIs (212, 213). Uveitis can be a complication of ICI treatment (214). Few cases of Vogt-Koyanagi-Harada disease have been described in melanoma patients, which hinted a possible cross-reactivity between T lymphocytes targeting melanoma cells and the melanocytes of the eye (215). In the cases of ICI-associated sicca syndrome, oral manifestations are more common than the ocular ones (216). Anti-Ro (SS-A) and anti-La (SS-B) are usually negative (216).

## Pre-Existing Autoimmune Disease and ICIs

Cancer patients with underlying autoimmune disease were initially excluded from ICI RCT (162, 217–219). Therefore, the prevalence and incidence of exacerbations of pre-existing autoimmune disorders was not immediately appreciated. Patients with autoimmune disease, however, represent 20 to 50 million people in the United States alone, and one study reported that approximately 13% of lung cancer patients had a concurrent diagnosis of autoimmune disease (220).

There is now evidence that irAEs are more frequent and occur faster in cancer patients with several autoimmune diseases (psoriasis, rheumatoid arthritis, IBD, systemic lupus erythematosus, vasculitis) (106–108). These findings suggest that a close monitoring is mandatory during early phases of treatment. The majority of autoimmune flares and irAEs can be managed without ICI discontinuation, but fatalities can occur (221). Conflicting results have been reported in studies concerning the risk of autoimmune flares or irAEs in patients with active *versus* inactive autoimmune disease (106, 108, 221, 222). Patients with underlying autoimmune disorders should be carefully managed by multidisciplinary teams.

## IrAEs and Efficacy of ICIs

In one large, retrospective study of ipilimumab, the treatment outcomes were similar in patients with and without irAEs (223). Subsequent studies reported that melanoma patients who develop vitiligo or endocrine complications have better tumor response and improved survival (122, 175, 224–226). The results of randomized trials have shown that patients who discontinue ICIs due to toxicity respond better than patients without irAEs (227). Patients that developed thyroiditis after PD-1 or PD-L1 blockade had longer overall survival compared to the thyroid irAE negative group (60, 225). Prospective studies are needed to verify whether different irAEs are associated with improved tumor response to ICIs.

## Management of IrAEs

No trials are evaluating the efficacy of different irAEs treatments. Therefore, management of irAEs are based on retrospective studies and expert consensus (11, 29, 31, 45, 174, 175, 228, 229). Low-grade irAEs usually do not need ICI discontinuation and immunosuppressive treatment. Higher grade irAEs may require both therapeutic strategies. **Table 2** schematically summarizes the anti-inflammatory and immunosuppressive drugs routinely used to treat ICI-related irAEs.

**Table 2:** Therapies used for immune adverse events associated with immune checkpoint inhibitors.

Drug	Mechanism of action	Efficacy in reversing irAE	Effect on tumor response	Clinical trial
Glucocorticoids	Anti-inflammatory and immunosuppressive	First line therapy for most ICI-induced irAEs Not effective for reversing endocrinopathies	Some studies suggest that response rates and survival are not affected by low doses of glucocorticoids (223, 224, 230), whereas others (122, 231–235) show negative impact of high doses. *In vitro* experiments data suggest negative impact of high dose glucocorticoids on anti-tumor effects of T cells (236).	
Infliximab	Anti-TNF-α mAb	Colitis (16).	Controversial results on the net effect of TNF-α inhibition on tumorigenesis (237–240). In murine models, prophylactic TNF-α inhibition eliminated ICI-induced colitis without affecting anti-tumor response (241).	NCT05034536: Comparison of pembrolizumab + infliximab *versus* pembrolizumab + placebo in patients with melanoma. NCT03293784: Comparison of infliximab or certolizumab + nivolumab + ipilimumab in patients with melanoma. NCT04407247: Comparison of vedolizumab *versus* infliximab for clinical remission/response of ICI-associated diarrhea/colitis.

Glucocorticoids are powerful anti-inflammatory and immunosuppressive drugs commonly employed as first-line treatment in patients with ICI-induced irAEs. The efficacy of glucocorticoids varies tremendously in the treatment of different types of irAEs. For instance, these compounds are effective in most cases but do not reverse hypophysitis, and high doses may worsen outcomes (122). By contrast, initial high-dose glucocorticoids were superior to intermediate and low-dose steroid in the treatment of ICI-associated myocarditis (242). Another important issue is the timing of initiation of glucocorticoid administration. In the above retrospective, observational study, early (≤ 24 hours) administration of glucocorticoids can also vary in different irAEs. For example, arthritis is also unique since inflammation often persists even after stopping ICIs and may require prolonged glucocorticoid treatment (40) and/or biological DMARDs (243).

Glucocorticoids are associated with potential multiple side effects and impact on anti-tumor response. Low doses of glucocorticoids used to treat irAEs do not affect the response rates and/or the survival of ICI-treated patients (223, 224, 230). A meta-analysis found that glucocorticoids do not negatively affect survival (230). However, glucocorticoids before or early during ICI treatment may negatively affect outcomes (231–234). Baseline treatment of lung cancer patients with anti-PD-1 and high doses (> 10 mg/day) of prednisone negatively affects outcomes compared to those treated with low dose glucocorticoids (231, 235). The side effects of glucocorticoids depend on the daily dose, the cumulative dose administered and possibly the type of underlying disease (244). Therefore, awareness of specific glucocorticoid-induced side effects is required.

irAEs refractory to glucocorticoids may require the administration of a mAb targeting TNF-α (i.e., infliximab) to treat certain irAEs such as colitis (16, 136, 237, 245) and pneumonitis (25, 175, 246). Preclinical studies suggest that prophylactic TNF-α inhibition eliminated ICI-induced colitis without affecting anti-tumor response (241). There is conflicting evidence on the effect of short-term TNF-α inhibition with infliximab to treat ICI-induced irAEs on overall survival in cancer patients (237–240). A comprehensive review has examined in detail the role of different cytokines in the pathophysiology of irAEs. In addition, the authors provided an in-depth analysis of strategies to uncouple the cytokine response that participates in ICI-associated irAEs (247).

## Potential Therapies and Prevention of IrAEs

The increasing use of ICIs in a growing number of solid and hematologic cancers requires us to offer the best-targeted therapies of irAEs. **Table 3** summarizes the potential therapies of irAEs.

**Table 3:** Emerging and potential future therapies for immune adverse events associated with immune checkpoint inhibitors.

Drug	Mechanism of action	Efficacy in reversing irAE	Effect on tumor response	Clinical trial
Vedolizumab	Anti-integrin α4β7 mAb	Colitis (137, 248). Prevention of autoimmune flares in patients with IBD (249).	Favorable clinical outcomes (248, 249).	NCT04407247: Comparison of vedolizumab *versus* infliximab for clinical response of ICI-induced diarrhea/colitis. NCT04797325: Comparison of vedolizumab *versus* prednisolone for clinical response of ICI-induced colitis.
Alemtuzumab	CD52 mAb	Myocarditis (198).	Unknown	
Abatacept	CTLA-4 agonist	Myocarditis (250).	Unknown	
Rituximab	Anti-CD20 mAb	Neurological complications (e.g., encephalitis and myasthenia gravis), bullous pemphigoid-like skin disease, renal vasculitis, hematological complications (12, 175, 204, 251–253).	Progression, partial and complete responses reported (251–253).	NCT03719131: Evaluation of rituximab on ICI-induced irAEs.
Tocilizumab	Anti-IL-6 receptor mAb	Pneumonitis, colitis, and pancreatitis (254). Inflammatory arthritis (255).	Clinical improvement with trend towards worse survival with increased doses of tocilizumab (254). 1/3 patients maintained anti-tumor response (255).	NCT03999749: Evaluation of tocilizumab on diarrhea and/or colitis and/or arthritis induced by ICIs. NCT04691817: Evaluation of tocilizumab on irAEs in patients with non-small lung cancer (NSCLC) treated with atezolizumab.
Secukinumab	Anti-IL-17A mAb	Psoriatic rash and colitis (256). Psoriasiform dermatological complication (257).	Tumor progression occurred in one patient (256). No impact on tumor response (257).	
Anakinra	IL-1 receptor antagonist	Experimental intestinal inflammation associated with combined immune checkpoint blockade in mice (96).	Unknown	NCT04576429: Evaluation of anakinra on irAEs and cytokine profile in patients with different cancers.
Fecal microbiota transplant	Possibly increased Tregs and decreased effector T cells (258).	Colitis (258).	Unknown	NCT04038619: Phase I trial of fecal microbiota transplant (FMT) for ICI-induced colitis/diarrhea. NCT04163289: Prevention of irAEs using fecal microbiota transplant. NCT03819296: Prevention of gastrointestinal irAEs by FMT in patients with melanoma or genitourinary cancer.
Certolizumab	anti-TNF-α			NCT03293784: Comparison of infliximab or certolizumab + nivolumab + ipilimumab in patients with melanoma.
Intravenous immunoglobulin (IVIG)				NCT04438382: Comparison of infliximab versus IVIG in patients with pneumonitis.

Understanding the pathophysiology of ICI-associated myocarditis and developing effective treatments is of great importance. The quest for novel therapies for glucocorticoid-resistant ICI myocarditis is a clinical unmet need. If symptoms and laboratory findings in ICI myocarditis do not regress upon high-dose glucocorticoids, other immunosuppressant agents [e.g. mycophenolate mofetil, methotrexate, calcineurin inhibitors, intravenous immunoglobulin (IVIG), anti-thymocyte globulin, rituximab and infliximab] may be considered for treatment of ICIs cardiotoxicity, but data are still controversial (201, 259, 260).

Alemtuzumab (anti-CD52 mAb) and abatacept, a protein consisting of the human CTLA-4 extracellular domain fused to the Fc portion of IgG, acting as a CTLA-4 agonist, have been employed for the treatment of single cases of glucocorticoid-refractory myocarditis (198, 250). Concerns with abatacept are possible infections and tumor progression. Abatacept has also been used to treat ICI-induced MG (261). Alemtuzumab causes T cell depletion and its impact on tumor growth remains unknown. Overall, the evidence available at present is insufficient to support any of the anecdotal, albeit reasonable, strategies outlined above and more evidence-based guidance in this critical care is urgently needed. While blocking TNF-α in heart failure (HF) has been proven contraindicated in symptomatic (NYHA III and IV) patients (262, 263), anti-TNF-α may be a promising approach to prevent the early stages of cardiotoxicity from anti-PD-1 immunotherapy (200).

Vedolizumab is a specific anti-integrin α4β7 antibody, used for the treatment of IBD (264–266). Preclinical studies have reported that vedolizumab induces remission in ICI-induced glucocorticoid-refractory colitis with good safety profiles (137, 248). Early treatment with vedolizumab is a potential treatment of ICI-associated colitis (138). Two clinical trials are evaluating the safety and efficacy of vedolizumab in the treatment of ICI-induced colitis (NCT04407247, NCT04797325).

Rituximab, a mAb targeting CD20, has been used in glucocorticoid-refractory encephalitis and myasthenia gravis (251, 252), bullous pemphigoid-like skin disease (12), renal vasculitis (253), and hematological complications (204).

Tocilizumab, a mAb anti-IL-6 receptor, has been used to treat ICI-associated arthritis (255) and glucocorticoid-refractory irAEs (254). The safety and efficacy of tocilizumab on ICI-associated irAEs are under evaluation in two clinical trials (NCT03999749, NCT04691817).

IL-17 blockade has been used in few patients with colon cancer and melanoma (256, 257). A recent study found that there is an upregulation of intestinal IL-1β in melanoma patients treated with CICB who developed high-grade colitis (96). In two mouse models, CICB was associated with intestinal inflammation characterized by upregulation of *Il1b*, but not *Tnfa* or *Il6*. Interestingly, mice concurrently treated with CICB and anakinra (anti-IL-1R) showed less intestinal inflammation. These parallel studies in humans and mice suggest that severe intestinal inflammation associated with CICB could be prevented by an IL-1R antagonist. A clinical trial is evaluating the safety and efficacy of anakinra on ICI-induced irAEs (NCT04576429).

Gut microbiome can influence efficacy of PD-1-based immunotherapy (103, 105). A clinical trial is prospectively analyzing the intestinal microbiome as predictor of ICI-associated irAEs (NCT04107311). Fecal microbiota transplant (FMT), which transfers an entire microbiome from a healthy donor to a recipient, is a therapeutic tool with several potential applications but numerous caveats (267). FMT may be a potential mechanism for treating ICI-induced colitis (258). FMT can be performed *via* an oral capsule containing fecal extracts (268–270), colonoscopy-guided transfer (269, 271) or enema (272, 273). Microbiome composition varies widely among healthy donors and can affects success rates (274, 275). FMT involves the risk for transmission of infectious agents *via* FMT (276). Regulatory authorities have released specific guidelines to offer FMT with safety at the time of COVID-19 pandemic (277). Two patients with refractory ICI-associated colitis were successfully treated with FMT (258). Clinical and experimental studies are needed to evaluate the efficacy of this approach as well as to provide further mechanistic insights. In summary, there is some evidence that microbiome manipulation could potentially impact cancer course and perhaps ICI-associated irAEs. Several clinical trials are evaluating the safety and efficacy of FMT on the prevention/treatment of intestinal irAEs (NCT04038619, NCT04163289, NCT03819296).

## Conclusions and Perspectives

Emerging real-world data suggests the incidence of ICI-associated irAEs may be higher than previously found in clinical trials. This trend has the potential to increase further as the use of canonical ICIs (anti-CTLA-4 and anti-PD-1/PD-L1), alone or in combination, is increasing exponentially and nearly 50% of patients treated will experience some form of irAEs (278). Furthermore, the combination of first and/or second generation of ICIs (e.g., anti-TIGIT) or with anti-angiogenic agents (279) could open a new scenario of irAEs associated with novel forms of cancer immunotherapy.

Great efforts have been devoted to the identification of genetic, humoral and cellular biomarkers predictive of irAEs associated with ICIs. Although these putative biomarkers have not been incorporated into clinical practice, they have highlighted some novel aspects of irAE pathogenesis. For instance, recent human and experimental studies have highlighted the contribution of specific gut microbiota to intestinal damage caused by CICB in melanoma patients (96). This study also suggests that specific peripheral blood signatures are associated with a risk of developing toxicity after CICB. Of course, additional studies of larger cohorts of ICI-treated patients with different cancers will be needed to validate these findings.

Management of irAEs is essentially based on retrospective studies and expert consensus (11, 29, 31, 45, 174, 175, 228, 229). Glucocorticoids, commonly employed as first-line treatment of irAEs, do not affect certain toxicities, and high doses may negatively affect outcomes (122, 231–234). Awareness of glucocorticoid-induced side effects is required. A wide spectrum of emerging therapeutic options, including mAbs (anti-TNF-α, anti-IL-6, anti-CD20, anti-IL-1R) (12, 96, 241, 251, 252, 254) and FMT (96, 258) are under clinical and experimental investigations. There is currently no clinical evidence that irAEs can be pharmacologically prevented. However, there is experimental evidence that prophylactic administration anti-TNF-α (241) or anti-IL-1R (96) is associated with less intestinal inflammation caused by CICB in different tumor models.

**Table 4** presents some of the outstanding pathophysiological and therapeutic questions that should be addressed to better understand the complexity of different irAEs. Due to the clinical complexity surrounding autoimmune disease development and management, Clinical Immunologists should be involved in the care of cancer patients before, during, and after checkpoint blockade. Perhaps, multidisciplinary irAE management teams, facilitating prompt diagnosis and treatment should be enlisted. Further research is needed to improve early diagnosis, understand immunological and genetic mechanisms, and develop management algorithms for these disorders.

**Table 4:** Outstanding pathophysiological and therapeutic questions relevant to immune-related adverse events (irAEs) associated with immune checkpoint inhibitors.

What are the immunological bases of different irAEs? Is there a genetic predisposition to explain why irAEs occur in some patients and not in others? Are genetic or epigenetic alterations linked to different irAEs? Are irAEs the new face of primary autoimmune disorders or rather a new disease entity? Why do some irAEs occur early whereas others occur late during ICI therapy? Can late-onset irAEs (i.e., accelerated atherosclerosis) up to several years after starting treatment occur? Are we accelerating atherosclerosis in cancer patients by releasing the brakes with ICIs? What is the long-term safety of cancer patients with pre-existing autoimmune disorders treated with ICIs? Are specific gut microbiota associated with different irAEs? Can gut microbiome be used to identify cancer patients who may develop irAEs? Is there a possibility of preventing irAEs with biological therapies (e.g., anti-TNF-α, anti-IL-1R)? Can gut microbiome engineering (e.g., fecal microbiota transplantation) lead to treatment of irAEs?	

## Author Contributions

RP, GC, FC, CT, and GV wrote and edited the manuscript. All authors contributed to the article and approved the submitted version.

## Funding

This work was supported in part by grants from the CISI-Lab Project (University of Naples Federico II) and TIMING Project and Campania Bioscience (Regione Campania).

## Conflict of Interest

The authors declare that the research was conducted in the absence of any commercial or financial relationships that could be construed as a potential conflict of interest.

## Publisher’s Note

All claims expressed in this article are solely those of the authors and do not necessarily represent those of their affiliated organizations, or those of the publisher, the editors and the reviewers. Any product that may be evaluated in this article, or claim that may be made by its manufacturer, is not guaranteed or endorsed by the publisher.

## Abbreviations

ACPA, anti-citrullinated peptide antibodies; *B. intestinalis*, *Bacteroides intestinalis*; CT, chest computed tomography; CICB, combined immune checkpoint blockade; CCP, cyclic citrullinated peptide; CTLA-4, cytotoxic T-lymphocyte-associated protein 4; DMARDs, disease modifying anti-rheumatic drugs; FMT, fecal microbiota transplant; G-CSF, granulocyte colony-stimulating factor; GM-CSF, granulocyte-macrophage colony-stimulating factor; GNAL, guanine nucleotide-binding protein G subunit alpha; HLA, human leukocyte antigen; ICIs, immune checkpoint inhibitors; irAEs, immune-related adverse events; IBD, inflammatory bowel disease; ITM2B, integral membrane protein 2B; IVIG), intravenous immunoglobulin therapy; MRI, magnetic resonance imaging; MACE, major adverse cardiac events; mAbs, monoclonal antibodies; MCP-1, monocyte chemoattractant protein-1; NCI-CTCAE, national cancer institute common terminology criteria for adverse events; NSCLC, non-small cell lung cancer; PD-1, programmed cell death protein 1; PD-L1, programmed cell death protein ligand 1; PRS, polygenic risk score; RCT, randomized controlled trial; RF, rheumatoid factor; SS-A, Sjogren’s syndrome antibodies anti-Ro; SS-B, Sjogren’s syndrome antibodies anti-La; TG, thyroglobulin; TPO, thyroid peroxidase; Trm, tissue-resident memory T; ^18^F-FDG, tomography/computed tomography with 2-[^18^F] fluorodeoxyglucose; TNF-α, tumor necrosis factor-α; T1D, type 1 diabetes.
